# Case Report: Neuropsychiatric improvement after treatment of pelvic venous disorder in a multisyndromic patient

**DOI:** 10.3389/fcvm.2025.1574432

**Published:** 2026-01-12

**Authors:** Steven J. Smith, Michael J. Sichlau, Peter C. Rowe, Dacre R. T. Knight, Brenda Chen, B. Holly Smith, Isabel C. Sichlau

**Affiliations:** 1 Department of Vascular and Interventional Radiology, Vascular and Interventional Professionals, LLC, Hinsdale, IL, United States; 2 Department of Pediatrics, The Johns Hopkins University, Baltimore, MD, United States; 3 Department of General Internal Medicine, The Mayo Clinic, Jacksonville, FL, United States; 4 College of Osteopathic Medicine, Touro University California, Vallejo, CA, United States; 5 Center for Advanced Study of Human Paleobiology, George Washington University, Washington, DC, United States; 6 Department of Psychology, Dietrich School of Arts and Sciences, University of Pittsburgh, Pittsburgh, PA, United States

**Keywords:** academic self-concept, allostatic overload, cerebral blood flow, chronic pelvic pain, medical imaging, mental health outcomes

## Abstract

**Objective:**

Venous Origin Chronic Pelvic Pain (VO-CPP) causes pelvic pain in women, and is associated with other dysautonomia syndromes such as postural orthostasis and tachycardia syndrome (POTS), chronic fatigue, interstitial cystitis, and fibromyalgia. These conditions have been shown to have associations with Ehlers–Danlos syndrome, migraine headache, irritable bowel syndrome, and brain fog. In this study, a multisyndromic patient was treated for VO-CPP. Pelvic pain, dysautonomia, and neuropsychiatric scores were tracked in this patient.

**Methods:**

In a multisyndromic patient with VO-CPP, abnormal venous pooling was treated with endovascular stenting and sclerotherapy. Neuropsychiatric testing was performed before and after intervention.

**Results:**

Along with improvement in scores for pelvic pain, POTS, interstitial cystitis, and migraines, repeat neuropsychiatric testing showed improvement in Memory Functioning Recall and Depression Inventory. The patient’s previous disability status was removed.

**Conclusions:**

The patient's response to endovascular treatment supports a unifying concept of diminished venous return as a contributor to multiple syndromes that may be linked to a single phenotype.

## Introduction

The chronic pelvic pain (CPP) condition known as “pelvic congestion syndrome (PCS)” (now commonly called Venous Origin Chronic Pelvic Pain, or “VO-CPP”) is responsible for causing up to 30% of chronic pelvic pain in women (1). Symptoms of VO-CPP typically include chronic positional pelvic pain and heaviness, vulvodynia, and dyspareunia. It can be safely and effectively treated by correcting pelvic venous drainage pathways to eliminate pelvic venous pooling. This treatment involves embolization of pelvic varices and/or stenting of compromised iliac vein outflow (2, 3). Recently, VO-CPP has been shown to be associated with other conditions of a pathophysiology of trapped blood volume below the waist, including “dysautonomia”-type symptoms such as postural orthostasis and tachycardia syndrome (POTS), and chronic fatigue syndrome (4, 5). Interstitial cystitis is also comorbid with dysautonomia and with pelvic varicosities (6). These conditions, in turn, are entwined with Ehlers–Danlos syndrome (EDS), migraine headache, and brain fog (5, 7–9).

A clinical challenge lies in considering a unifying concept of pathophysiology that spans multiple medical disciplines. In this study, we present a case of a complex multisystemic process in a woman referred for CPP and then treated for VO-CPP. Her response to treatment alleviated not only her CPP but also brain fog.

## Case presentation

A 42-year-old Caucasian woman was referred to an outpatient clinic with CPP. Her neurologist, who was treating her for migraine headaches, had noted symptoms of CPP. She suffered from a multitude of comorbidities as outlined below, involving not only gynecologic and neurologic systems, but also gastrointestinal, musculoskeletal, cardiovascular, endocrine, hematologic, and psychiatric systems.

*Gynecologic*: The patient was treated by gynecology for chronic vulvodynia and dyspareunia.

*Neurologic*: The patient was managed by neurology for chronic migraines as well as seizure disorder. She had numerous Emergency Department admissions for severe migraines requiring intravenous treatment, leading to opioid dependence. Her headache neurologist noted worsening confusion and ability to concentrate during clinic visits, and the clinician ordered neuropsychiatric testing (Supplementary Table 1). While her intelligence test result was in the Superior range, she was inattentive, and her short-term memory was placed in the 47th percentile. The impression was that she had attention deficit disorder (ADD), following which accommodations for college were advised. She was completely disabled; she dropped out of graduate school and was unable to drive due to severe disorientation. She was on multiple medications (Supplementary Table 2), but even after the list of medications was reduced, she reported limited ability to concentrate.

*Gastrointestinal*: History and records noted a long history of severe constipation and bloating, with a diagnosis of irritable bowel syndrome (IBS). A precipitous decline in her health status was marked by a spontaneous colon perforation 8 years prior. Ever since she suffered from this perforation, she underwent a total of 13 abdominal surgeries, including total colectomy. However, her surgeons noted “poor healing”.

*Musculoskeletal*: She was treated for back pain related to lumbar spondylosis and underwent several spine surgeries for this, as well as for a spontaneous cerebrospinal fluid (CSF) leak.

*Cardiovascular*: She was seen by cardiology and neurology for POTS with frequent traumatic syncopal episodes.

*Endocrine*: She was also undergoing treatment by endocrinology for pituitary failure, hypothyroidism, and adrenal insufficiency.

*Hematologic*: She suffered a pulmonary embolus related to the thrombus around a peripherally inserted central catheter (PICC). The PICC was placed due to difficult venous access for her frequent intravenous migraine treatments.

*Psychiatric*: She was undergoing treatment for anxiety and depression.

She had no history of electrolyte imbalance, and her renal function was normal. Because of her extensive and multisystemic ailments, she had contacts of 60 doctors in her phone.

At consultation, she was noted to be a poor historian, and she demonstrated confusion and distraction. She reported daily migraine headaches, difficulty concentrating, as well as non-cyclic pelvic pain associated with upright posture for the past 8 years. She also reported vulvodynia and dyspareunia for the same time period. Questionnaire responses to the International Pelvic Pain Society (IPSS) Pelvic Pain Assessment Form documented chronic pelvic pain (International Pelvic Pain Society, 2007), and responses to the validated Orthostatic Hypotension Questionnaire (OHQ) qualified as “moderate to extreme” orthostatic hypotension (10). A physical examination showed a tall and muscular woman who was anxious and confused. She often trailed off her speech and did not complete sentences. She had a Beighton score exceeding the score needed to diagnose generalized joint hypermobility (11, 12). An external pelvic examination showed exquisite vulvar tenderness and scattered vulvar varicose veins.

**Figure 1:**
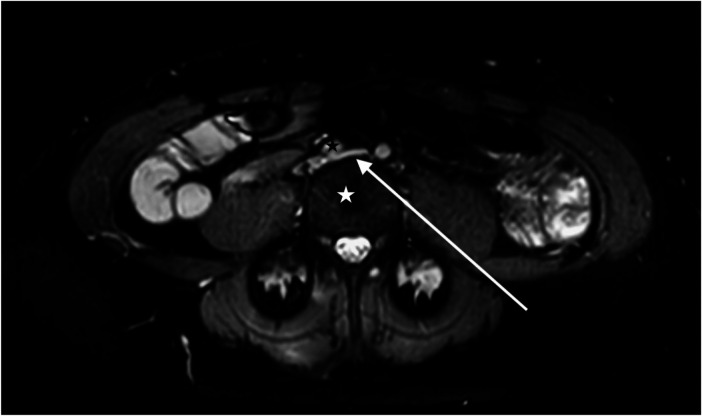
A magnetic resonance venogram in the axial plane shows a compressed left common iliac vein (white arrow) between the lumbar 4 vertebral body (white star) and the right common iliac artery (black star).

Cross-sectional imaging with magnetic resonance venography (Figure 1) revealed >50% diameter compression of the left common iliac vein between the right common iliac artery and the lumbar vertebral body.

The impressions from the consultation were as follows: (1) VO-CPP shown by her IPSS score, a history of >6 months non-cyclic pelvic pain, and an iliac vein stenosis of >50%; (2) orthostatic hypotension shown by her OHQ score; (3) refractory migraine headaches; (4) Ehlers–Danlos syndrome (generalized joint hypermobility subtype) related to her Beighton test and poor healing history, and (5) brain fog. Based on these clinical impressions and cross-sectional imaging findings, it was recommended that she undergo venography with intravascular ultrasound (IVUS) and possible iliac vein stenting.

**Figure 2:**
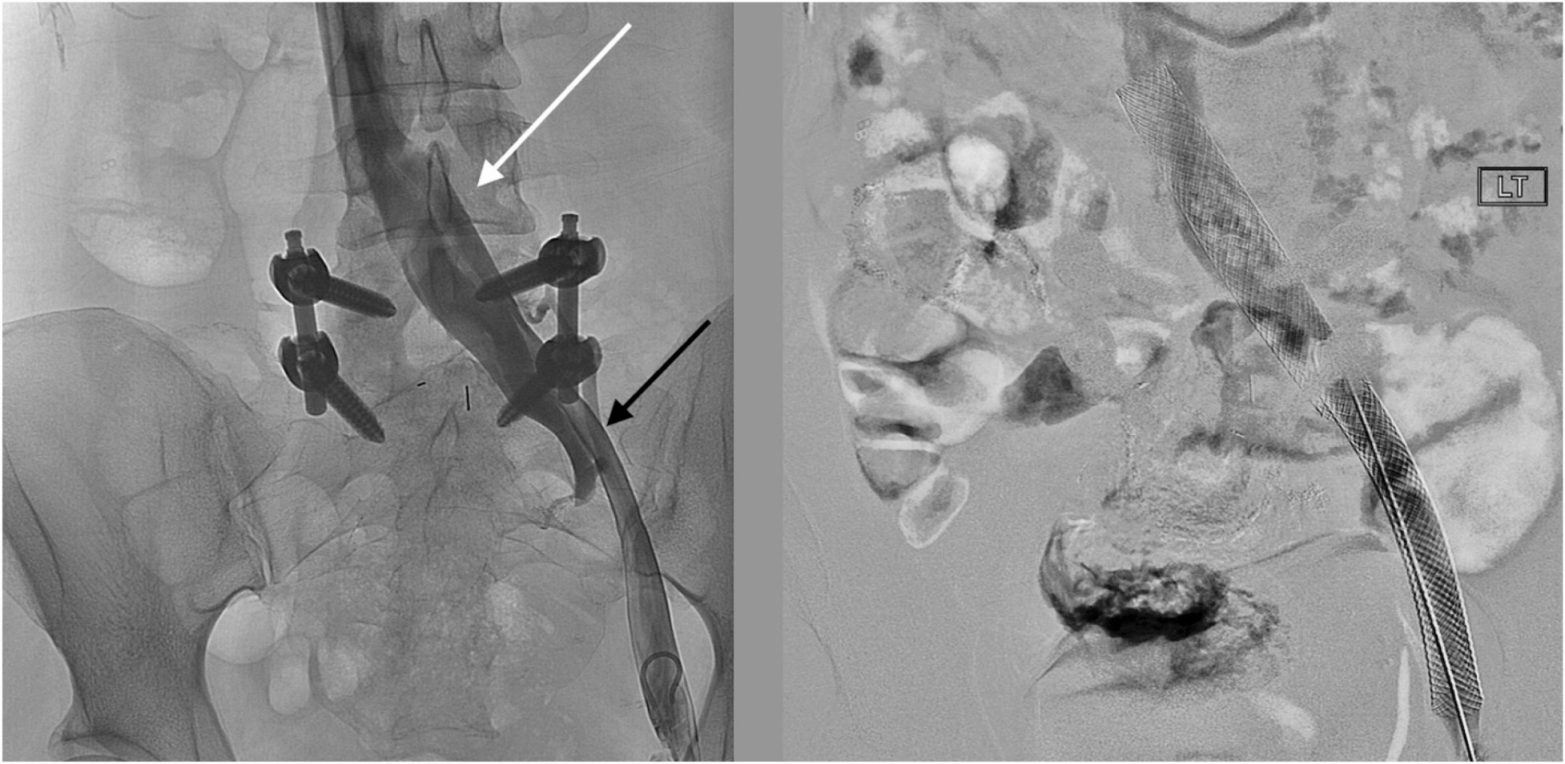
Venographic images at the time of procedure from a left common femoral vein access show (left) an en-face widening of the central left common iliac vein (white arrow) and the stenotic left external iliac vein (black arrow). After stenting (right), the affected areas are covered by the self-expanding stents.

**Figure 3:**
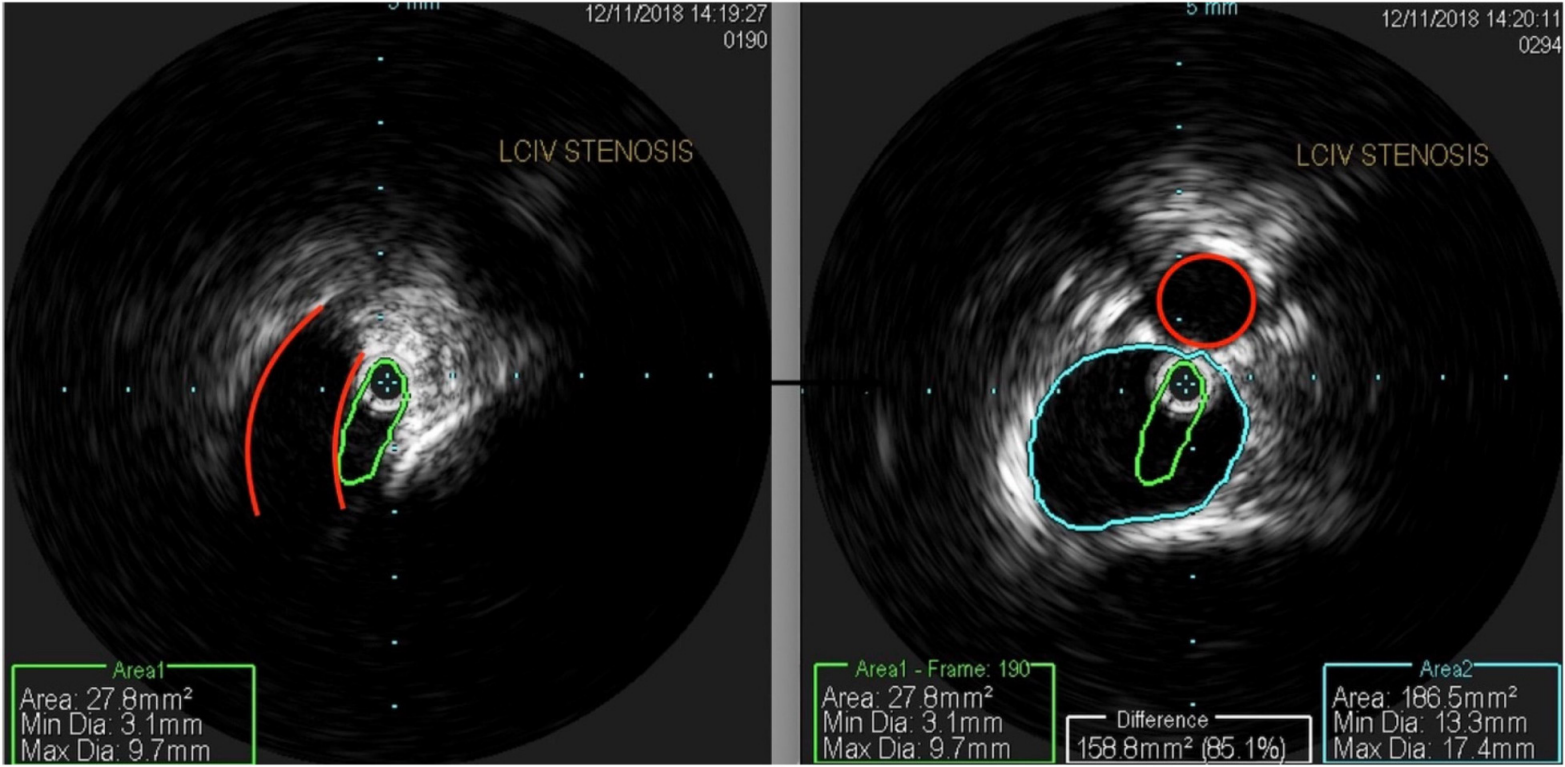
An intravascular ultrasound shows (left) a profile of a compressed left common iliac vein (green) by an overlying right common iliac artery (red). The right-sided image shows a composite of the normal portion of the left common iliac vein (blue) and a compressed central left common iliac vein (green), representing 85% area stenosis. The right common iliac artery is outlined in red.

**Figure 4:**
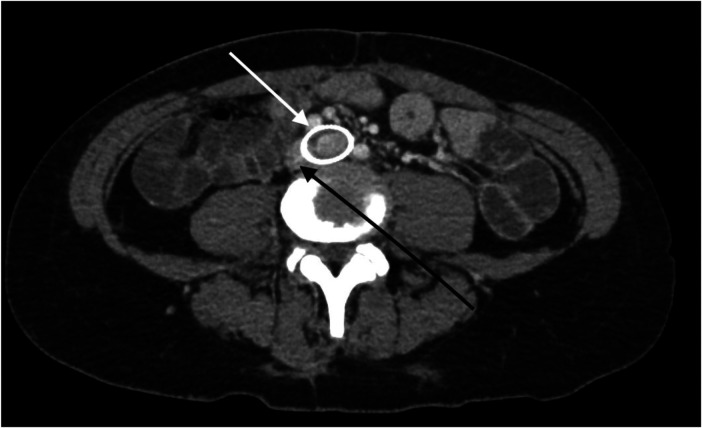
A CT venogram in the axial plane shows a stented left common iliac vein (white arrow) and the adjacent compressed right common iliac vein (black arrow).

The patient underwent treatment at AMITA Hinsdale Hospital (Hinsdale, IL, USA). Under moderate sedation, the patient underwent traditional transcatheter venography (Figure 2). This, along with intravascular ultrasound (IVUS), confirmed significant (25) left common iliac vein stenosis by an overlying common iliac artery of greater than 50% (25) (Figure 3). She was initially treated with placement of a left iliac vein stent. Post procedure, she was prescribed clopidogrel for 30 days. 30–40 mm Hg waist-high compression hose were ordered, and she was advised to hydrate daily with 100 oz of electrolyte drinks. She underwent a routine clinical and imaging follow-up at 1 and 6 months. Confusion and dizzy spells were reported as alleviated at 1 month, but still some diminished cognitive clarity persisted. At 6 months, she reported improvement in cognition to a point where she reenrolled in graduate studies and resumed driving. According to her neurologist, she was having significantly fewer headaches and fatigue was reported to be reduced. Slight dizziness persisted, and she was started on a short course of midodrine. Dyspareunia persisted. She was subsequently administered a percutaneous ultrasound-guided injection of the vaginal wall and vulvar varices with 0.5% sotradecol foam using the Tessari technique (13). There were no immediate procedural complications, and her dyspareunia resolved after 3 months. At 12 months after iliac vein stenting, repeat neuropsychiatric testing (with the same examiner) showed her memory at the 95th percentile. Her full-scale IQ increased modestly. The Beck Depression Inventory II score moved to the normal range. The overall impression was “normal exam” (Supplementary Table 1). At 18 months after stenting, her dizziness returned, and dyspareunia reappeared. An ultrasound suggested a developing right iliac vein stenosis. Subsequently, a CT venogram showed the left stent pressing upon an already partially compressed right common iliac vein (Figure 4). She then underwent placement of a right-sided iliac vein stent. She was again prescribed clopidogrel, but for only 3 months, and again advised to drink 100 oz electrolyte solution daily and wear waist-high compression hose.

At 30-month follow-up after initial iliac vein stenting, she reported no dyspareunia and no vaginal pain. She experienced much-reduced migraine headaches, which were down to four per month from every day. She had no syncopal episodes ever since she underwent her procedures. After completing graduate school, she started her own business. Many of her preprocedural medications were discontinued (Supplementary Table 2). Upon an examination, she appeared more alert than her preprocedural condition. Her speech was spontaneous and she completed sentences. She had no vulvar tenderness or vulvar varicose veins. She was scheduled for an annual clinic and imaging follow-up.

## Discussion

This patient case illustrates how pelvic venous obstruction or insufficiency may be related to other sometimes disabling syndromes of unknown cause. POTS and its resulting orthostatic hypotension can be disabling (8). While POTS certainly has multifactorial potential causes, in this patient, we propose that it responded to addressing impaired venous blood return to the heart, with resulting diminished cerebral blood flow. Physiologic studies have shown that patients subjected to orthostatic stress had decreased cardiac filling pressure and that female patients showed statistically significant drops in stroke volume and systolic blood pressure, and increased heart rate. It has also been shown that sympathetic response to orthostatic positional changes in terms of muscle sympathetic nerve activity and serum catecholamine measurements was not significantly different between males and females, supporting the conclusion that orthostatic intolerance in females is due to the cardiovascular components of decreased cardiac filling pressure and stroke volume, as opposed to a blunted sympathetic response (14). A study by Edgell et al. took this concept further and tied in the other concepts with cerebral perfusion. In this study, patients subjected to orthostatic stress in terms of moving from a supine to an upright position also showed significant increases in heart rate and decreases in stroke volume. Furthermore, the same patients were measured to have significant decreases in cardiac output, end-tidal CO_2_, and middle cerebral artery velocity with orthostatic stress (15).

In the patient presented in our study, we propose that her cognitive symptoms had at least some basis in decreased cerebral perfusion as described by Edgell et al. Prior to endovascular treatment, her imaging showed an obstruction of venous flow in the pelvis due to vascular compression. With the correction of this vascular compression, venous congestion of the pelvic structures diminished, cardiac venous return likely improved, and measured cognitive function improved. Future studies that could lend support to this hypothesis would involve controlled measurement of cardiac output and cerebral arterial flow both before and after endovascular treatment.

In our practice, we administer a survey of comorbid symptoms to all VO-CPP patients at each visit. Many of our patients have been found to qualify as having severe dysautonomia based on validated criteria. Symptoms of POTS and migraines have been alleviated significantly in many after vein treatment (5). A survey of the PCS online support group done in 2021 was positive for correlation with many of these related groups of comorbidities (POTS, IBS, migraine, interstitial cystitis, chronic fatigue, anxiety attacks) (16). An alternative term to consider for this constellation of symptoms and syndromes may be Orthostatic Flow Syndrome.

Patients with chronic fatigue, POTS, and fibromyalgia experience decreased blood flow to the brain (9, 11, 17, 18) and therefore vascularly linked. In addition, these patients often report the symptom of brain fog. Brain fog has been ill-defined in the past but is often described by patients as cloudiness of thought patterns, impaired memory, difficulty thinking and focusing, and/or difficulty communicating. These descriptors are altogether different from brain fog reports by patients with general fatigue, anxiety, or depression (i.e., thoughts moving too quickly, detached, lost, sleepy) (19), suggesting that the definition of brain fog symptoms is not translated across disorders. Cognitive function has been shown to decline following orthostatic stress in adults with myalgic encephalomyelitis/chronic fatigue syndrome (ME/CFS) (26). Furthermore, patients with POTS experience heightened cognitive impairment, specifically with working memory, accuracy, and information processing, when undergoing orthostatic stress (20). Patients with impaired cerebral blood flow perform poorly on cognitive challenges testing short-term memory and alertness when compared with healthy individuals (21). Decreased cerebral blood flow has been correlated with brain fog symptoms, and what has been regarded as autonomic nervous system dysfunction could relate to functional hypovolemia because of venous pooling in the pelvis leading to decreased cardiac venous return and resulting diminished cerebral perfusion as described by Edgell et al. (15).

In the literature, inattention/brain fog has sometimes been diagnosed as adult attention-deficit/hyperactivity disorder (ADHD) or has been reported as a characteristic of dysautonomia and EDS (22, 23). A recently published series found that among patients presenting with chronic pelvic pain, 76% of them also reported brain fog as a symptom in questionnaire responses. After venous intervention, repeat administration of the same questionnaires showed that their mean mean 0–10 brain fog scores decreased by 59% (5). Based on greater than 6-month symptom history and formal neuropsychiatric testing, the patient in this study would be categorized as adult ADHD, Predominantly Inattentive Subtype (24). Our impression was that she could have been suffering cognitively because of diminished cerebral blood flow.

## Conclusion

In this study, a complex patient case was presented with the treatment of chronic pelvic pain using stents and sclerosis of the abnormal veins. The patient’s pelvic pain was relieved, as were disabling migraines, syncope, and brain fog, originally diagnosed as ADD. The patient's disability status was removed. While other factors such as reduced pain including reduced migraine headache frequency, reduced need for pain medication, improved sleep, and overall diminished polypharmacy could also be contributory to this patient's symptom alleviation, the potential relevance of compromised cardiac venous return in the setting of chronic venous disease is discussed in this report in this study. While a logical physiologic explanation was submitted for this patient's improved neuropsychiatric testing after correcting for impaired pelvic venous return, the single-case nature of this study and a lack of long-term follow-up beyond 30 months restrict our ability to draw generalizations. A further study using randomization techniques, quantitative venous flow measurement of cerebral perfusion, and serum catecholamine measurements before and after venous treatment may help solidify the concepts introduced here.

## Data Availability

The original contributions presented in the study are included in the article/Supplementary Material, and further inquiries can be directed to the corresponding author.
