# Supplementary material for: Case Report: Neuropsychiatric improvement after treatment of pelvic venous disorder in a multisyndromic patient
Source: Front Cardiovasc Med. 2026 Jan 12;12:1574432. doi: 10.3389/fcvm.2025.1574432 (PMC12833758; doi:10.3389/fcvm.2025.1574432)
Supplement: Supplementary file 1 [file Table1.pdf]

Supplementary Table 1. Neuropsychiatric Testing Summary

Test	Result Pre Treatment (percentile)	Result Post Treatment (percentile)
Victoria Symptom Validity Test	Valid	Valid
National Adult Reading Test (NART)	above average. FSIQ=112	above average. FSIQ=118
Repeatable Battery for Assessment of Neuropsychiatric Status (RBANS)–		
Total score	107 (68)	109 (73)
Language Index Score	above average. 112 (79)	average. 101 (53)
Verbal Fluency	average. T score 47	average. T score 51
Visuospatial/Constructional	average. 105 (63)	average. 105 (63)
Memory Functioning acquisition	112 (79)	109 (73)
**Memory Functioning recall**	**99 (47)**	**121 (92)**
Attention Index Score	average. 100 (50)	average. 100 (50)
Controlled Word Association (COWA)		
Verbal Fluency	below expectation. T score 38	average. T score 48
Connors Continuous Performance Test - 3rd Edition (CPT-3)		
Omission errors	T score 47	T score 90
Comission errors	T score 45	T score 64
Mean reaction time	T score 61	T score 68
Response variability	T score 51	T score 88
Trail Making Test A	superior. T score 65	above average. T score 56
Trail Making Test B	above average. T score 55	average. T score 51
Wisconsin Card Sorting Test	T score 57	T score 51
California Verbal Learning Test II (CVLT II)	average. T score 54	above average. T score 64
**Beck Depression Inventory II (BDI-II)**	**moderate. T score 72 (96)**	**no evidence. T score 42 (22)**

Behavior Rating Inventory of Executive Function - Adult (BRIEF-A). Lower scores indicate improvement		
Inhibit	54 (79)	48 (53)
Shift	43 (39)	39 (25)
Emotional control	38 (20)	38 (20)
Self-Monitoring	46 (52)	37 (20)
Initiation	57 (85)	57 (85)
Working Memory	78 (99)	60 (84)
Planning and Organization	75 (99)	53 (67)
Task Monitoring	69 (98)	56 (76)
Organization of Materials	60 (88)	54 (70)
Overall Quality of Life Percentile	93	99
