# Supplementary material for: Case Report: Neuropsychiatric improvement after treatment of pelvic venous disorder in a multisyndromic patient
Source: Front Cardiovasc Med. 2026 Jan 12;12:1574432. doi: 10.3389/fcvm.2025.1574432 (PMC12833758; doi:10.3389/fcvm.2025.1574432)
Supplement: Supplementary file 2 [file Table2.pdf]

Supplementary Table 2. Medication List at Presentation

Etelcalcitide 70 mg SQ monthly
Dessicated Thyroid 90 mg daily
Lorcaserin XR 20 mg daily
Biotin 5000 mcg SL daily
Bupropion 150 mg daily
Candesartan 8 mg daily
Desvenlafaxine 50 mg daily
Erenumab-aooe
Esketamine nasal spray 84mg twice a week
Fetzima 80 mg daily
Hydrocortisone 10mg qAM, 5mg qPM
Lisdexamfetamine 30 mg daily
Memantine 10 mg TID
Metformin 500 mg daily
Midodrine 5 mg TID
Naproxen 500 mg twice a day
Naratriptan 2.5 mg daily
Ondansetron 4 mg ODT daily
Pramipexole 1 mg daily
Progesterone 100 mg daily
Spironolactone 100 mg po twice a day
Topiramate 150 mg daily

Medications at 1 year follow-up

Lorcaserin XR 20 mg daily - same
Biotin 5000 mcg SL daily - same
Bupropion 300 mg daily – increased
Esketamine nasal spray 84mg twice a week - same
Fetzima 80 mg daily – same
Hydrocortisone 10mg qAM, 5mg qPM - same
Lisdexamfetamine 30 mg daily – increased to 70 mg daily
Memantine 10 mg TID - same
Metformin 500 mg daily - same
Ondansetron 4 mg ODT daily - same
Acarbose 25 mg daily – new
Aripiprazole 2 mg daily - new
Famcyclovir 250 mg daily – new
Metoclopramide 10 mg daily – new
Rimegepant 75 mg ODT daily – new
Semaglutide 0.25 mg SQ weekly – new

Discontinued: Etelcalcitide, Dessicated thyroid, Candesartan, Desvenlafaxine, Erenumab, Midodrine, Naproxen, Naratriptan, Pramipexole, Progesterone, Spironolactone
